# Supplementary material for: Time Trends in Prevalence and Antimicrobial Resistance of Respiratory Pathogens in a Tertiary Hospital in Rome, Italy: A Retrospective Analysis (2018–2023)
Source: Antibiotics (Basel). 2025 Sep 15;14(9):932. doi: 10.3390/antibiotics14090932 (PMC12466844; doi:10.3390/antibiotics14090932)
Supplement: Supplementary file 1 [file antibiotics-14-00932-s001.zip › antibiotics-3839032-supplementary.pdf]

Supplementary Materials - Table S1. Microorganism/drug associations trends over time.

Organism	Drugs	Break points	Break points	R (%)	IC95 (R%)	Pearson Coef.$^{\gamma}$	β coef.$^{\delta}$	IC95 β coef.$^{\varepsilon}$	p-value	R^2
A. baumannii	amikacin	S ≤ 8	R ≥ 16	85.9	82.3; 88.9	-0.442	-1.616	-3.296; 0.064	0.058	0.195
A. baumannii	ciprofloxacin	I ≤ 1	R ≥ 2	93.9	91.3; 95.9	-0.532	-0.863	-1.565; -0.16	0.019	0.283
A. baumannii	colistin	S ≤ 2	R ≥ 4	2.6	1.4; 4.6	-0.52	-0.59	-1.086; -0.094	0.023	0.27
A. baumannii	doripenem	I ≤ 2	R ≥ 4	100	46.3; 100	n.a.	n.a.	n.a.	n.a.	n.a.
A. baumannii	gentamicin	S ≤ 4	R ≥ 8	90.3	87.1; 92.7	-0.349	-0.621	-1.474; 0.232	0.143	0.122
A. baumannii	imipenem	S ≤ 2	R ≥ 8	91.8	88.0; 94.5	-0.579	-0.941	-1.619; -0.263	0.009	0.335
A. baumannii	levofloxacin	S ≤ 0.5	R ≥ 2	96.6	87.3; 99.4	-0.421	-0.692	-1.586; 0.201	0.118	0.178
A. baumannii	meropenem	S ≤ 2	R ≥ 16	93.3	90.5; 95.3	-0.496	-0.794	-1.504; -0.083	0.031	0.246
A. baumannii	tobramycin	S ≤ 4	R ≥ 8	89.8	85.7; 92.9	-0.501	-1.061	-2.07; -0.053	0.04	0.251
A. baumannii	trimethoprim/sulfamethoxazole	S ≤ 2	R ≥ 8	89.1	85.8; 91.8	-0.284	-0.486	-1.325; 0.354	0.239	0.081
C. albicans	amphotericinb	S ≤ 1	R ≥ 2	0	0.0; 8.0	n.a.	n.a.	n.a.	n.a.	n.a.
C. albicans	anidulafungin	S ≤ 0.016	R ≥ 0.03	11.8	2.1; 37.7	0.141	0.58	-2.483; 3.644	0.678	0.02
C. albicans	fluconazole	S ≤ 2	R ≥ 8	0	0.0; 8.0	n.a.	n.a.	n.a.	n.a.	n.a.
C. albicans	itraconazole	S ≤ 0.064	R ≥ 0.12	0	0.0; 22.9	n.a.	n.a.	n.a.	n.a.	n.a.
C. albicans	micafungin	S ≤ 0.032	R ≥ 0.06	0	0.0; 25.3	n.a.	n.a.	n.a.	n.a.	n.a.
C. albicans	posaconazole	S ≤ 0.064	R ≥ 0.12	0	0.0; 80.2	n.a.	n.a.	n.a.	n.a.	n.a.
C. albicans	voriconazole	S ≤ 0.064	R ≥ 0.5	0	0.0; 43.9	n.a.	n.a.	n.a.	n.a.	n.a.
C. glabrata	amphotericinb	S ≤ 1	R ≥ 2	0	0.0; 20.0	n.a.	n.a.	n.a.	n.a.	n.a.
C. glabrata	anidulafungin	S ≤ 0.064	R ≥ 0.12	0	0.0; 20.9	n.a.	n.a.	n.a.	n.a.	n.a.
C. glabrata	fluconazole	I ≤ 16	R ≥ 32	10.5	1.8; 34.5	-0.703	-4.095	-8.231; 0.042	0.052	0.494
C. glabrata	micafungin	S ≤ 0.032	R ≥ 0.06	0	0.0; 25.3	n.a.	n.a.	n.a.	n.a.	n.a.
C. tropicalis	amphotericinb	S ≤ 1	R ≥ 2	0	0.0; 34.5	n.a.	n.a.	n.a.	n.a.	n.a.
C. tropicalis	anidulafungin	S ≤ 0.064	R ≥ 0.12	0	0.0; 43.9	n.a.	n.a.	n.a.	n.a.	n.a.
C. tropicalis	fluconazole	S ≤ 2	R ≥ 8	10	0.5; 45.9	-0.693	-4.575	-8.828; -0.323	0.038	0.48
C. tropicalis	itraconazole	S ≤ 0.125	R ≥ 0.25	14.3	0.8; 58.0	-0.758	-6.154	-12.244; -0.064	0.048	0.574
C. tropicalis	micafungin	S ≤ 0.064	R ≥ 0.12	0	0.0; 48.3	n.a.	n.a.	n.a.	n.a.	n.a.

C. tropicalis	posaconazole	S ≤ 0.064	R ≥ 0.12	20	1.1; 70.1	-0.814	-8.29	-19.145; 2.564	0.093	0.663
C. tropicalis	voriconazole	S ≤ 0.125	R ≥ 0.5	0	0.0; 40.2	n.a.	n.a.	n.a.	n.a.	n.a.
E. cloacae	amikacin	S ≤ 8	R ≥ 16	0	0.0; 4.5	n.a.	n.a.	n.a.	n.a.	n.a.
E. cloacae	amoxicillin/clavulanicacid	S ≤ 8	R ≥ 16	92.9	85.3; 96.8	-0.422	-0.796	-1.672; 0.079	0.072	0.178
E. cloacae	aztreonam	S ≤ 1	R ≥ 8	40	7.3; 83.0	-0.655	-16.667	-261.198; 227.864	0.546	0.429
E. cloacae	cefepime	S ≤ 1	R ≥ 8	5	1.8; 11.7	-0.273	-0.63	-1.766; 0.506	0.258	0.074
E. cloacae	cefotaxime	S ≤ 1	R ≥ 4	23.3	15.1; 33.8	0.14	0.508	-1.395; 2.411	0.579	0.02
E. cloacae	ceftazidime/avibactam	S ≤ 8	R ≥ 16	0	0.0; 5.4	n.a.	n.a.	n.a.	n.a.	n.a.
E. cloacae	ceftazidime	S ≤ 1	R ≥ 8	26.2	18.3; 36.0	0.031	0.112	-1.737; 1.961	0.9	0.001
E. cloacae	ceftolozane/tazobactam	S ≤ 2	R ≥ 4	16.7	9.7; 26.7	-0.261	-1.369	-4.158; 1.42	0.312	0.068
E. cloacae	ceftriaxone	S ≤ 1	R ≥ 4	41.7	16.5; 71.4	0.827	10.57	0.611; 20.529	0.042	0.685
E. cloacae	ciprofloxacin	S ≤ 0.25	R ≥ 1	7.8	3.7; 15.3	-0.266	-0.795	-2.268; 0.677	0.27	0.071
E. cloacae	colistin	S ≤ 2	R ≥ 4	3.9	1.3; 10.3	-0.09	-0.134	-0.886; 0.619	0.713	0.008
E. cloacae	ertapenem	S ≤ 0.5	R ≥ 1	9.7	2.5; 26.9	-0.434	-1.135	-2.698; 0.429	0.138	0.188
E. cloacae	gentamicin	S ≤ 2	R ≥ 4	4.9	1.8; 11.5	-0.212	-0.495	-1.665; 0.675	0.384	0.045
E. cloacae	imipenem	S ≤ 2	R ≥ 8	0	0.0; 6.1	n.a.	n.a.	n.a.	n.a.	n.a.
E. cloacae	levofloxacin	S ≤ 0.5	R ≥ 2	5.9	0.3; 30.8	-0.549	-3.765	-8.89; 1.361	0.126	0.301
E. cloacae	meropenem	S ≤ 2	R ≥ 16	0	0.0; 4.5	n.a.	n.a.	n.a.	n.a.	n.a.
E. cloacae	piperacillin/tazobactam	S ≤ 8	R ≥ 16	22.1	14.5; 32.0	-0.161	-0.599	-2.48; 1.283	0.511	0.026
E. cloacae	piperacillin	S ≤ 8	R ≥ 16	40	7.3; 83.0	-0.655	-16.667	-261.198; 227.864	0.546	0.429
E. cloacae	tobramycin	S ≤ 2	R ≥ 4	6.9	2.6; 16.1	-0.427	-2.036	-4.51; 0.438	0.099	0.182
E. cloacae	trimethoprim/sulfamethoxazole	S ≤ 2	R ≥ 8	7.8	3.7; 15.2	-0.316	-0.924	-2.343; 0.495	0.187	0.1
E. coli	amikacin	S ≤ 8	R ≥ 16	3.5	1.8; 6.5	-0.234	-0.273	-0.856; 0.309	0.336	0.055
E. coli	amoxicillin/clavulanicacid	S ≤ 8	R ≥ 16	52.7	46.4; 58.8	-0.315	-1.057	-2.686; 0.571	0.189	0.099
E. coli	ampicillin/sulbactam	S ≤ 8	R ≥ 16	100	5.5; 100	n.a.	n.a.	n.a.	n.a.	n.a.
E. coli	ampicillin	S ≤ 8	R ≥ 16	0	0.0; 94.5	n.a.	n.a.	n.a.	n.a.	n.a.
E. coli	aztreonam	S ≤ 1	R ≥ 8	36	18.7; 57.4	0.351	4.357	-11.794; 20.509	0.496	0.123
E. coli	cefepime	S ≤ 1	R ≥ 8	27.7	22.6; 33.5	-0.462	-1.193	-2.363; -0.022	0.046	0.214

E. coli	cefotaxime	S ≤ 1	R ≥ 4	41.4	34.8; 48.4	-0.457	-2.002	-3.998; -0.006	0.049	0.209
E. coli	cefpodoxime	S ≤ 1	R ≥ 2	100	5.5; 100	n.a.	n.a.	n.a.	n.a.	n.a.
E. coli	ceftazidime/avibactam	S ≤ 8	R ≥ 16	0.9	0.2; 3.7	-0.043	-0.032	-0.411; 0.347	0.861	0.002
E. coli	ceftazidime	S ≤ 1	R ≥ 8	35.5	30.1; 41.4	-0.438	-0.96	-1.968; 0.048	0.061	0.192
E. coli	ceftolozane/tazobactam	S ≤ 2	R ≥ 4	4.2	2.1; 8.2	0.246	0.294	-0.298; 0.887	0.309	0.061
E. coli	ceftriaxone	S ≤ 1	R ≥ 4	43.1	29.6; 57.7	-0.226	-1.46	-5.42; 2.499	0.437	0.051
E. coli	cefuroxime	S ≤ 8	R ≥ 16	100	5.5; 100	n.a.	n.a.	n.a.	n.a.	n.a.
E. coli	ciprofloxacin	S ≤ 0.25	R ≥ 1	48.2	42.2; 54.2	-0.531	-1.888	-3.431; -0.346	0.019	0.282
E. coli	colistin	S ≤ 2	R ≥ 4	0.4	0; 2.2	0.301	0.044	-0.027; 0.115	0.21	0.091
E. coli	doripenem	S ≤ 1	R ≥ 4	0	0.0; 94.5	n.a.	n.a.	n.a.	n.a.	n.a.
E. coli	ertapenem	S ≤ 0.5	R ≥ 1	0.8	0; 5.1	-0.258	-0.07	-0.205; 0.064	0.286	0.067
E. coli	fosfomycin	S ≤ 8	R ≥ 16	8.4	4.6; 14.5	0.146	0.312	-0.772; 1.397	0.551	0.021
E. coli	gentamicin	S ≤ 2	R ≥ 4	20.2	15.8; 25.4	-0.627	-1.706	-2.792; -0.621	0.004	0.393
E. coli	imipenem	S ≤ 2	R ≥ 8	0.6	0; 3.7	0.387	0.088	-0.019; 0.195	0.101	0.15
E. coli	levofloxacin	S ≤ 0.5	R ≥ 2	53.2	41.6; 64.6	-0.101	-0.577	-3.483; 2.329	0.681	0.01
E. coli	meropenem	S ≤ 2	R ≥ 16	0.7	0.1; 2.8	0.094	0.026	-0.116; 0.169	0.702	0.009
E. coli	nitrofurantoin	S ≤ 64	R ≥ 128	1.9	0.1; 11.4	0.241	0.769	-1.181; 2.719	0.407	0.058
E. coli	norfloxacin	S ≤ 0.5	R ≥ 1	100	5.5; 100	n.a.	n.a.	n.a.	n.a.	n.a.
E. coli	piperacillin/tazobactam	S ≤ 8	R ≥ 16	13.1	9.5; 17.7	-0.38	-0.757	-1.701; 0.186	0.109	0.144
E. coli	piperacillin	S ≤ 8	R ≥ 16	80.8	60.0; 92.7	0.297	0.938	-2.534; 4.409	0.518	0.088
E. coli	tigecycline	S ≤ 0.5	R ≥ 1	1.6	0.3; 6.3	0.125	0.078	-0.238; 0.394	0.609	0.016
E. coli	tobramycin	S ≤ 2	R ≥ 4	15.3	10.4; 22.0	-0.251	-0.757	-2.306; 0.791	0.315	0.063
E. coli	trimethoprim/sulfamethoxazole	S ≤ 2	R ≥ 8	39.7	34.1; 45.7	-0.451	-1.353	-2.723; 0.017	0.053	0.203
H. influenzae	amoxicillin/clavulanicacid	S ≤ 2	R ≥ 4	6.9	1.2; 24.2	-0.03	-0.106	-2.353; 2.14	0.92	0.001
H. influenzae	ampicillin	S ≤ 1	R ≥ 2	13.3	4.4; 31.6	0.036	0.179	-2.795; 3.154	0.898	0.001
H. influenzae	azithromycin	S ≤ 4	R ≥ 8	0	0.0; 14.1	n.a.	n.a.	n.a.	n.a.	n.a.
H. influenzae	cefotaxime	S ≤ 0.125	R ≥ 0.25	3.4	0.2; 19.6	0.141	0.322	-1.036; 1.679	0.617	0.02
H. influenzae	ceftriaxone	S ≤ 0.125	R ≥ 0.25	0	0.0; 14.1	n.a.	n.a.	n.a.	n.a.	n.a.
H. influenzae	ciprofloxacin	S ≤ 0.032	R ≥ 0.06	10.7	2.8; 29.4	0.335	1.737	-1.334; 4.807	0.241	0.112
H. influenzae	clarithromycin	S ≤ 32	R ≥ 64	0	0.0; 14.6	n.a.	n.a.	n.a.	n.a.	n.a.

H. influenzae	doxycycline	S ≤ 1	R ≥ 2	10	2.6; 27.7	0.018	0.084	-2.763; 2.93	0.95	n.a.
H. influenzae	erythromycin	S ≤ 16	R ≥ 32	0	0.0; 14.6	n.a.	n.a.	n.a.	n.a.	n.a.
H. influenzae	levofloxacin	S ≤ 0.064	R ≥ 0.12	10.3	2.7; 28.5	0.352	1.777	-1.055; 4.61	0.198	0.124
H. influenzae	meropenem	S ≤ 2	R ≥ 4	0	0.0; 14.1	n.a.	n.a.	n.a.	n.a.	n.a.
H. influenzae	tetracycline	S ≤ 2	R ≥ 4	0	0.0; 14.6	n.a.	n.a.	n.a.	n.a.	n.a.
H. influenzae	trimethoprim/sulfamethoxazole	S ≤ 0.5	R ≥ 2	37.9	21.3; 57.6	-0.133	-1.086	-5.942; 3.769	0.637	0.018
K. aerogenes	amikacin	S ≤ 8	R ≥ 16	1.4	0.1; 8.9	-0.387	-0.526	-1.167; 0.115	0.101	0.15
K. aerogenes	amoxicillin/clavulanicacid	S ≤ 8	R ≥ 16	95.4	86.2; 98.8	-0.157	-0.351	-1.477; 0.775	0.52	0.025
K. aerogenes	ampicillin/sulbactam	S ≤ 8	R ≥ 16	100	5.5; 100	n.a.	n.a.	n.a.	n.a.	n.a.
K. aerogenes	aztreonam	S ≤ 1	R ≥ 8	50	9.2; 90.8	0.811	4.11	-33.575; 41.794	0.398	0.658
K. aerogenes	cefepime	S ≤ 1	R ≥ 8	4.5	1.2; 13.4	-0.246	-1.053	-3.173; 1.068	0.31	0.061
K. aerogenes	cefotaxime	S ≤ 1	R ≥ 4	29.6	18.4; 43.8	-0.04	-0.243	-3.468; 2.982	0.875	0.002
K. aerogenes	ceftazidime/avibactam	S ≤ 8	R ≥ 16	1.9	0.1; 11.2	-0.539	-1.296	-2.509; -0.083	0.038	0.291
K. aerogenes	ceftazidime	S ≤ 1	R ≥ 8	29	19.0; 41.3	0.08	0.474	-2.54; 3.488	0.744	0.006
K. aerogenes	ceftolozane/tazobactam	S ≤ 2	R ≥ 4	14.8	7.1; 27.7	-0.194	-0.771	-3.107; 1.566	0.489	0.038
K. aerogenes	ceftriaxone	S ≤ 1	R ≥ 4	30	8.1; 64.6	0.654	5.513	-1.809; 12.835	0.111	0.428
K. aerogenes	ciprofloxacin	S ≤ 0.25	R ≥ 1	2.9	0.5; 11.2	-0.177	-0.263	-1.011; 0.485	0.468	0.031
K. aerogenes	colistin	S ≤ 2	R ≥ 4	0	0.0; 6.6	n.a.	n.a.	n.a.	n.a.	n.a.
K. aerogenes	doripenem	S ≤ 1	R ≥ 4	100	5.5; 100	n.a.	n.a.	n.a.	n.a.	n.a.
K. aerogenes	ertapenem	S ≤ 0.5	R ≥ 1	8	1.4; 27.5	-0.171	-0.836	-4.032; 2.36	0.576	0.029
K. aerogenes	gentamicin	S ≤ 2	R ≥ 4	2.9	0.5; 11.0	-0.177	-0.263	-1.011; 0.485	0.468	0.031
K. aerogenes	imipenem	S ≤ 2	R ≥ 8	2.2	0.1; 13.2	-0.343	-1.647	-4.482; 1.187	0.229	0.118
K. aerogenes	levofloxacin	S ≤ 0.5	R ≥ 2	0	0.0; 25.3	n.a.	n.a.	n.a.	n.a.	n.a.
K. aerogenes	meropenem	S ≤ 2	R ≥ 16	2.9	0.5; 11.0	-0.215	-0.877	-2.914; 1.16	0.376	0.046
K. aerogenes	piperacillin/tazobactam	S ≤ 8	R ≥ 16	27.3	17.4; 39.8	-0.089	-0.543	-3.655; 2.57	0.718	0.008
K. aerogenes	piperacillin	S ≤ 8	R ≥ 16	50	9.2; 90.8	0.811	4.11	-33.575; 41.794	0.398	0.658
K. aerogenes	tobramycin	S ≤ 2	R ≥ 4	2.3	0.1; 13.5	0.378	0.385	-0.241; 1.011	0.203	0.143
K. aerogenes	trimethoprim/sulfamethoxazole	S ≤ 2	R ≥ 8	2.9	0.5; 11.0	-0.177	-0.263	-1.011; 0.485	0.468	0.031
K. oxytoca	amikacin	S ≤ 8	R ≥ 16	1.9	0.1; 11.4	0.213	0.145	-0.222; 0.513	0.412	0.045
K. oxytoca	amoxicillin/clavulanicacid	S ≤ 8	R ≥ 16	2.2	0.1; 13.0	0.413	0.47	-0.218; 1.158	0.161	0.17

K. oxytoca	aztreonam	S ≤ 1	R ≥ 8	14.3	0.8; 58.0	-0.707	-20	-56.748; 16.748	0.182	0.5
K. oxytoca	cefepime	S ≤ 1	R ≥ 8	4	0.7; 14.9	-0.361	-1.483	-3.596; 0.629	0.155	0.13
K. oxytoca	cefotaxime	S ≤ 1	R ≥ 4	0	0.0; 14.6	n.a.	n.a.	n.a.	n.a.	n.a.
K. oxytoca	ceftazidime/avibactam	S ≤ 8	R ≥ 16	2.1	0.1; 12.7	-0.397	-1.629	-3.7; 0.442	0.114	0.158
K. oxytoca	ceftazidime	S ≤ 1	R ≥ 8	3.8	0.7; 14.1	-0.361	-1.483	-3.596; 0.629	0.155	0.13
K. oxytoca	ceftolozane/tazobactam	S ≤ 2	R ≥ 4	4.3	0.7; 15.7	-0.361	-1.483	-3.596; 0.629	0.155	0.13
K. oxytoca	ceftriaxone	S ≤ 1	R ≥ 4	0	0.0; 22.9	n.a.	n.a.	n.a.	n.a.	n.a.
K. oxytoca	ciprofloxacin	S ≤ 0.25	R ≥ 1	0	0.0; 8.4	n.a.	n.a.	n.a.	n.a.	n.a.
K. oxytoca	colistin	S ≤ 2	R ≥ 4	1.9	0.1; 11.4	0.387	0.317	-0.099; 0.734	0.125	0.15
K. oxytoca	ertapenem	S ≤ 0.5	R ≥ 1	0	0.0; 17.8	n.a.	n.a.	n.a.	n.a.	n.a.
K. oxytoca	gentamicin	S ≤ 2	R ≥ 4	1.9	0.1; 11.4	0.213	0.145	-0.222; 0.513	0.412	0.045
K. oxytoca	imipenem	S ≤ 2	R ≥ 8	0	0.0; 14.1	n.a.	n.a.	n.a.	n.a.	n.a.
K. oxytoca	levofloxacin	S ≤ 0.5	R ≥ 2	4.2	0.2; 23.1	0.27	0.399	-0.455; 1.253	0.331	0.073
K. oxytoca	meropenem	S ≤ 2	R ≥ 16	0	0.0; 8.4	n.a.	n.a.	n.a.	n.a.	n.a.
K. oxytoca	piperacillin/tazobactam	S ≤ 8	R ≥ 16	1.9	0.1; 11.4	-0.397	-1.629	-3.7; 0.442	0.114	0.158
K. oxytoca	piperacillin	S ≤ 8	R ≥ 16	42.9	11.8; 79.8	-0.756	-20	-51.824; 11.824	0.139	0.571
K. oxytoca	tobramycin	S ≤ 2	R ≥ 4	0	0.0; 14.1	n.a.	n.a.	n.a.	n.a.	n.a.
K. oxytoca	trimethoprim/sulfamethoxazole	S ≤ 2	R ≥ 8	1.9	0.1; 11.4	-0.397	-1.629	-3.7; 0.442	0.114	0.158
K. pneumoniae	amikacin	S ≤ 8	R ≥ 16	11.1	8.8; 13.9	-0.04	-0.053	-0.739; 0.632	0.871	0.002
K. pneumoniae	amoxicillin/clavulanicacid	S ≤ 8	R ≥ 16	72	68.0; 75.7	-0.221	-0.43	-1.4; 0.54	0.363	0.049
K. pneumoniae	amoxicillin	S ≤ 8	R ≥ 16	100	5.5; 100	n.a.	n.a.	n.a.	n.a.	n.a.
K. pneumoniae	ampicillin/sulbactam	S ≤ 8	R ≥ 16	100	5.5; 100	n.a.	n.a.	n.a.	n.a.	n.a.
K. pneumoniae	aztreonam	S ≤ 1	R ≥ 8	69.4	56.2; 80.1	0.732	3.613	1.598; 5.628	0.002	0.536
K. pneumoniae	cefepime	S ≤ 1	R ≥ 8	66.8	62.8; 70.5	0.195	0.378	-0.594; 1.349	0.423	0.038
K. pneumoniae	cefiderocol	S ≤ 2	R ≥ 4	38.5	15.1; 67.7	-0.763	-13.48	-29.335; 2.374	0.078	0.582
K. pneumoniae	cefotaxime	S ≤ 1	R ≥ 4	63.4	57.5; 68.9	-0.406	-1.054	-2.268; 0.159	0.084	0.165
K. pneumoniae	ceftazidime/avibactam	S ≤ 8	R ≥ 16	11.5	9.0; 14.6	0.601	0.963	0.308; 1.618	0.006	0.361
K. pneumoniae	ceftazidime	S ≤ 1	R ≥ 8	69.4	65.6; 73.0	0.211	0.403	-0.554; 1.361	0.387	0.044
K. pneumoniae	ceftibuten	S ≤ 1	R ≥ 2	100	5.5; 100	n.a.	n.a.	n.a.	n.a.	n.a.
K. pneumoniae	ceftolozane/tazobactam	S ≤ 2	R ≥ 4	53.5	49.1; 57.8	0.594	1.895	0.583; 3.207	0.007	0.353

K. pneumoniae	ceftriaxone	S ≤ 1	R ≥ 4	80.3	74.9; 84.8	0.485	1.299	-0.173; 2.772	0.079	0.235
K. pneumoniae	ciprofloxacin	S ≤ 0.25	R ≥ 1	67.6	63.6; 71.3	0.179	0.267	-0.486; 1.021	0.464	0.032
K. pneumoniae	colistin	S ≤ 2	R ≥ 4	3.4	2.2; 5.3	-0.575	-0.381	-0.659; -0.103	0.01	0.33
K. pneumoniae	doripenem	S ≤ 1	R ≥ 4	0	0.0; 94.5	n.a.	n.a.	n.a.	n.a.	n.a.
K. pneumoniae	ertapenem	S ≤ 0.5	R ≥ 1	58.8	53.5; 64.0	0.61	2.072	0.695; 3.449	0.006	0.372
K. pneumoniae	gentamicin	S ≤ 2	R ≥ 4	33	29.3; 36.9	-0.478	-1.352	-2.623; -0.081	0.038	0.229
K. pneumoniae	imipenem	S ≤ 2	R ≥ 8	31	25.7; 36.9	0.056	0.164	-1.331; 1.659	0.82	0.003
K. pneumoniae	levofloxacin	S ≤ 0.5	R ≥ 2	70.3	65.0; 75.1	0.568	1.641	0.424; 2.858	0.011	0.323
K. pneumoniae	meropenem/vaborbactam	S ≤ 8	R ≥ 16	0	0.0; 26.8	n.a.	n.a.	n.a.	n.a.	n.a.
K. pneumoniae	meropenem	S ≤ 2	R ≥ 16	44.2	40.3; 48.3	0.28	0.757	-0.569; 2.084	0.245	0.079
K. pneumoniae	moxifloxacin	S ≤ 0.25	R ≥ 0.5	100	5.5; 100	n.a.	n.a.	n.a.	n.a.	n.a.
K. pneumoniae	ofloxacin	S ≤ 0.25	R ≥ 1	100	5.5; 100	n.a.	n.a.	n.a.	n.a.	n.a.
K. pneumoniae	piperacillin/tazobactam	S ≤ 8	R ≥ 16	62.4	58.3; 66.3	0.174	0.341	-0.645; 1.327	0.476	0.03
K. pneumoniae	piperacillin	S ≤ 8	R ≥ 16	82	69.6; 90.2	0.742	1.988	0.913; 3.064	0.002	0.551
K. pneumoniae	tobramycin	S ≤ 2	R ≥ 4	33.1	27.5; 39.2	-0.315	-1.487	-3.783; 0.809	0.19	0.099
K. pneumoniae	trimethoprim/sulfamethoxazole	S ≤ 2	R ≥ 8	63	59.0; 66.8	0.224	0.434	-0.529; 1.397	0.356	0.05
P. aeruginosa	amikacin	S ≤ 16	R ≥ 32	7.9	6.1; 10.2	-0.089	-0.106	-0.718; 0.506	0.719	0.008
P. aeruginosa	aztreonam	I ≤ 16	R ≥ 32	23.2	17.6; 29.9	0.376	0.927	-0.243; 2.097	0.113	0.141
P. aeruginosa	cefepime	I ≤ 8	R ≥ 16	28.3	25.1; 31.7	-0.018	-0.04	-1.158; 1.078	0.941	n.a.
P. aeruginosa	cefiderocol	S ≤ 2	R ≥ 4	25	8.3; 52.6	-0.656	-9.148	-19.651; 1.355	0.077	0.431
P. aeruginosa	ceftazidime/avibactam	S ≤ 8	R ≥ 16	17.4	14.5; 20.7	-0.117	-0.267	-1.425; 0.891	0.633	0.014
P. aeruginosa	ceftazidime	I ≤ 8	R ≥ 16	37.7	34.2; 41.3	-0.003	-0.008	-1.291; 1.275	0.99	n.a.
P. aeruginosa	ceftolozane/tazobactam	S ≤ 4	R ≥ 8	17.4	14.5; 20.7	-0.232	-0.659	-2.072; 0.755	0.339	0.054
P. aeruginosa	ciprofloxacin	I ≤ 00.5	R ≥ 1	33.6	30.3; 37.2	-0.373	-1.018	-2.313; 0.277	0.116	0.139
P. aeruginosa	colistin	S ≤ 4	R ≥ 8	0.9	0.4; 2.0	-0.276	-0.086	-0.239; 0.067	0.253	0.076
P. aeruginosa	doripenem	I ≤ 2	R ≥ 4	80	29.9; 98.9	n.a.	n.a.	n.a.	1	n.a.
P. aeruginosa	imipenem	I ≤ 4	R ≥ 8	35.3	31.5; 39.3	-0.219	-0.591	-1.937; 0.755	0.367	0.048
P. aeruginosa	levofloxacin	I ≤ 2	R ≥ 4	30.9	25.2; 37.1	0.06	0.214	-1.598; 2.027	0.806	0.004
P. aeruginosa	meropenem/vaborbactam	S ≤ 8	R ≥ 16	77.8	40.2; 96.1	-0.065	-1.25	-36.757; 34.257	0.918	0.004
P. aeruginosa	meropenem	S ≤ 2	R ≥ 16	29.6	26.4; 33.1	-0.248	-0.636	-1.909; 0.637	0.306	0.061

P. aeruginosa	piperacillin/tazobactam	I ≤ 16	R ≥ 32	47.2	43.5; 50.9	0.325	0.611	-0.3; 1.522	0.175	0.105
P. aeruginosa	piperacillin	I ≤ 16	R ≥ 32	44.1	37.1; 51.4	0.403	1.276	-0.207; 2.759	0.087	0.162
P. aeruginosa	tobramycin	S ≤ 2	R ≥ 4	15.4	12.6; 18.6	-0.355	-0.881	-2.068; 0.305	0.136	0.126
P. mirabilis	amikacin	S ≤ 8	R ≥ 16	20.2	12.9; 30.0	-0.025	-0.147	-3.184; 2.89	0.92	0.001
P. mirabilis	amoxicillin/clavulanicacid	S ≤ 8	R ≥ 16	53.4	42.5; 64.0	-0.126	-0.74	-3.827; 2.347	0.618	0.016
P. mirabilis	aztreonam	S ≤ 1	R ≥ 8	28.6	5.1; 69.7	0.344	2.066	-5.773; 9.905	0.505	0.118
P. mirabilis	cefepime	S ≤ 1	R ≥ 8	11.7	6.3; 20.4	0.039	0.172	-2.097; 2.44	0.875	0.001
P. mirabilis	cefotaxime	S ≤ 1	R ≥ 4	47.4	35.9; 59.1	-0.354	-2.248	-5.522; 1.025	0.164	0.125
P. mirabilis	ceftazidime/avibactam	S ≤ 8	R ≥ 16	0	0.0; 6.7	n.a.	n.a.	n.a.	n.a.	n.a.
P. mirabilis	ceftazidime	S ≤ 1	R ≥ 8	34.7	25.5; 45.3	-0.026	-0.146	-3.051; 2.759	0.917	0.001
P. mirabilis	ceftolozane/tazobactam	S ≤ 2	R ≥ 4	13.2	6.6; 24.1	0.01	0.054	-3.16; 3.268	0.972	n.a.
P. mirabilis	ceftriaxone	S ≤ 1	R ≥ 4	41.7	16.5; 71.4	-0.157	-3.175	-23.153; 16.804	0.711	0.025
P. mirabilis	ciprofloxacin	S ≤ 0.25	R ≥ 1	61.1	50.5; 70.7	-0.071	-0.427	-3.496; 2.642	0.773	0.005
P. mirabilis	colistin	S ≤ 2	R ≥ 4	98.9	93.4; 99.9	-0.387	-0.158	-0.35; 0.034	0.101	0.15
P. mirabilis	ertapenem	S ≤ 0.5	R ≥ 1	0	0.0; 11.2	n.a.	n.a.	n.a.	n.a.	n.a.
P. mirabilis	gentamicin	S ≤ 2	R ≥ 4	41.1	31.2; 51.6	-0.281	-1.74	-4.779; 1.299	0.244	0.079
P. mirabilis	imipenem	S ≤ 2	R ≥ 8	17.2	9.0; 29.9	-0.219	-0.975	-3.459; 1.509	0.414	0.048
P. mirabilis	levofloxacin	S ≤ 0.5	R ≥ 2	52.6	29.5; 74.8	0.052	0.52	-6.461; 7.502	0.871	0.003
P. mirabilis	meropenem	S ≤ 2	R ≥ 16	0	0.0; 4.8	n.a.	n.a.	n.a.	n.a.	n.a.
P. mirabilis	piperacillin/tazobactam	S ≤ 8	R ≥ 16	4.2	1.4; 11.0	0.217	0.459	-0.599; 1.518	0.373	0.047
P. mirabilis	piperacillin	S ≤ 8	R ≥ 16	57.1	20.2; 88.2	0.42	3.306	-6.613; 13.225	0.407	0.176
P. mirabilis	tobramycin	S ≤ 2	R ≥ 4	35.7	23.7; 49.7	0.054	0.368	-3.894; 4.629	0.854	0.003
P. mirabilis	trimethoprim/sulfamethoxazole	S ≤ 2	R ≥ 8	56.8	46.3; 66.8	-0.005	-0.035	-3.308; 3.239	0.982	n.a.
S. aureus	cefoxitin	S ≤ 4	R ≥ 8	38.5	15.1; 67.7	-0.392	-4.759	-15.901; 6.383	0.336	0.154
S. aureus	ceftaroline	S ≤ 1	R ≥ 4	0	0.0; 1.4	n.a.	n.a.	n.a.	n.a.	n.a.
S. aureus	ceftobiprole	S ≤ 2	R ≥ 4	16.7	2.9; 49.1	0.405	6.311	-10.064; 22.686	0.367	0.164
S. aureus	ciprofloxacin	I ≤ 2	R ≥ 4	0	0.0; 94.5	n.a.	n.a.	n.a.	n.a.	n.a.
S. aureus	clindamycin	S ≤ 0.25	R ≥ 0.5	5.8	4.2; 8.0	-0.239	-0.257	-0.793; 0.278	0.325	0.057
S. aureus	dalbavancin	S ≤ 0.25	R ≥ 0.5	25	6.7; 57.2	-0.879	-13.35	-21.669; -5.03	0.009	0.773
S. aureus	daptomycin	S ≤ 1	R ≥ 2	0.3	0.1; 1.2	-0.26	-0.093	-0.271; 0.084	0.282	0.068

S. aureus	doxycycline	S ≤ 1	R ≥ 2	15.4	2.7; 46.3	0.283	3.209	-7.645; 14.062	0.497	0.08
S. aureus	erythromycin	S ≤ 1	R ≥ 2	58.2	54.4; 62.0	-0.128	-0.243	-1.208; 0.721	0.601	0.016
S. aureus	fusidicacid	S ≤ 1	R ≥ 2	3.1	2.0; 4.8	-0.42	-0.198	-0.417; 0.021	0.073	0.177
S. aureus	gentamicin	S ≤ 2	R ≥ 4	4	2.7; 5.9	-0.106	-0.07	-0.406; 0.266	0.665	0.011
S. aureus	levofloxacin	I ≤ 1	R ≥ 2	58.7	54.9; 62.5	-0.707	-1.564	-2.365; -0.763	0.001	0.499
S. aureus	linezolid	S ≤ 4	R ≥ 8	0	0.0; 0.7	n.a.	n.a.	n.a.	n.a.	n.a.
S. aureus	moxifloxacin	S ≤ 0.25	R ≥ 0.5	41.7	16.5; 71.4	-0.464	-7.039	-22.507; 8.429	0.295	0.215
S. aureus	nitrofurantoin	S ≤ 64	R ≥ 128	0	0.0; 28.3	n.a.	n.a.	n.a.	n.a.	n.a.
S. aureus	oxacillin	S ≤ 2	R ≥ 4	58.4	54.5; 62.1	-0.734	-1.596	-2.351; -0.84	n.a.	0.539
S. aureus	penicilling	S ≤ 0.125	R ≥ 0.25	87.5	84.6; 89.9	-0.641	-0.872	-1.406; -0.338	0.003	0.411
S. aureus	quinupristin/dalfopristin	S ≤ 1	R ≥ 2	0	0.0; 94.5	n.a.	n.a.	n.a.	n.a.	n.a.
S. aureus	rifampin	S ≤ 0.064	R ≥ 0.12	2.7	1.7; 4.3	-0.638	-0.392	-0.634; -0.15	0.003	0.407
S. aureus	tedizolid	S ≤ 0.5	R ≥ 1	8.3	0.4; 40.2	0.402	0.971	-1.569; 3.511	0.371	0.162
S. aureus	teicoplanin	S ≤ 2	R ≥ 4	1	0.5; 2.2	0.548	0.149	0.032; 0.266	0.015	0.3
S. aureus	tetracycline	S ≤ 1	R ≥ 2	6	4.3; 8.1	-0.176	-0.17	-0.657; 0.316	0.47	0.031
S. aureus	tigecycline	S ≤ 0.5	R ≥ 1	0.2	0; 1.0	-0.344	-0.05	-0.12; 0.02	0.149	0.119
S. aureus	trimethoprim/sulfamethoxazole	S ≤ 2	R ≥ 8	0.9	0.4; 2.0	-0.1	-0.041	-0.25; 0.168	0.682	0.01
S. aureus	vancomycin	S ≤ 2	R ≥ 4	0	0.0; 0.7	n.a.	n.a.	n.a.	n.a.	n.a.
S. maltophilia	trimethoprim/sulfamethoxazole	I ≤ 2	R ≥ 4	10.4	5.4; 18.7	0.533	1.083	0.203; 1.963	0.019	0.284
S. marcescens	amikacin	S ≤ 8	R ≥ 16	2.6	0.5; 10.0	0.079	0.175	-0.961; 1.312	0.749	0.006
S. marcescens	amoxicillin/clavulanicacid	S ≤ 8	R ≥ 16	85.1	73.8; 92.2	-0.333	-1.192	-2.919; 0.536	0.164	0.111
S. marcescens	aztreonam	S ≤ 1	R ≥ 8	11.1	0.6; 49.3	-0.098	-1.429	-45.753; 42.896	0.902	0.01
S. marcescens	cefepime	S ≤ 1	R ≥ 8	5.3	1.7; 13.8	-0.052	-0.132	-1.414; 1.151	0.831	0.003
S. marcescens	cefotaxime	S ≤ 1	R ≥ 4	5.2	1.3; 15.3	-0.241	-1.029	-3.229; 1.17	0.336	0.058
S. marcescens	ceftazidime/avibactam	S ≤ 8	R ≥ 16	1.8	0.1; 10.6	-0.351	-0.765	-1.887; 0.357	0.167	0.123
S. marcescens	ceftazidime	S ≤ 1	R ≥ 8	13.2	6.8; 23.3	-0.251	-0.939	-2.791; 0.914	0.3	0.063
S. marcescens	ceftolozane/tazobactam	S ≤ 2	R ≥ 4	10.5	4.4; 22.2	-0.233	-0.845	-2.789; 1.099	0.369	0.054
S. marcescens	ceftriaxone	S ≤ 1	R ≥ 4	44.4	15.3; 77.3	0.468	6.406	-7.506; 20.319	0.29	0.219
S. marcescens	ciprofloxacin	S ≤ 0.25	R ≥ 1	6.6	2.4; 15.3	-0.406	-2.281	-4.905; 0.344	0.084	0.165
S. marcescens	colistin	S ≤ 2	R ≥ 4	96.1	88.1; 99.0	0.154	0.329	-0.752; 1.41	0.529	0.024

S. marcescens	ertapenem	S ≤ 0.5	R ≥ 1	7.1	1.2; 25.0	0.621	3.885	0.631; 7.138	0.023	0.386
S. marcescens	gentamicin	S ≤ 2	R ≥ 4	3.9	1.0; 11.9	0.221	0.526	-0.66; 1.713	0.363	0.049
S. marcescens	imipenem	S ≤ 2	R ≥ 8	2.1	0.1; 12.7	-0.38	-0.845	-2.14; 0.449	0.18	0.144
S. marcescens	levofloxacin	S ≤ 0.5	R ≥ 2	16.7	4.4; 42.3	0.271	1.86	-3.114; 6.833	0.42	0.074
S. marcescens	meropenem	S ≤ 2	R ≥ 16	1.3	0.1; 8.1	-0.301	-0.409	-1.072; 0.254	0.21	0.091
S. marcescens	piperacillin/tazobactam	S ≤ 8	R ≥ 16	28.6	14.0; 48.9	0.094	0.727	-4.688; 6.142	0.771	0.009
S. marcescens	piperacillin	S ≤ 8	R ≥ 16	66.7	30.9; 91.0	0.153	2.857	-53.329; 59.043	0.847	0.023
S. marcescens	tobramycin	S ≤ 2	R ≥ 4	16.7	8.0; 30.8	0.57	1.567	0.145; 2.989	0.033	0.325
S. marcescens	trimethoprim/sulfamethoxazole	S ≤ 2	R ≥ 8	1.3	0.1; 8.1	0.344	0.351	-0.139; 0.841	0.149	0.119

$^{\gamma}$: Pearson coefficient, statistical index that measures the strength and direction of the linear relationship between two variables; $^{\delta}$: regression coefficient, statistical index that measures how the dependent variable (number of standardized isolates) changes when the independent variable (quarter - time) varies by one unit; $^{\varepsilon}$: 95% confidence interval of the β regression coefficient (inf, sup),
